# Age Inclusivity in Higher Education: Ensuring Excellence for Students, Faculty, and Staff

**DOI:** 10.1093/geroni/igaf122.2143

**Published:** 2025-12-31

**Authors:** Jacqueline Eaton, Rebekah Perkins, Nina Silverstein

## Abstract

Age inclusivity has positive outcomes for learning, including improving health, reducing social isolation, and decreasing ageism. Yet in higher education, age-stratification is often reinforced in how we view traditional vs nontraditional students and role expectations based on individual definitions of age. This symposium explores an initiative to assess age-friendly practices and beliefs and iteratively develop opportunities to improve educational excellence by addressing age-friendly practices. Eaton et al., discuss the use of the Age-Friendly Inventory and Campus Climate Survey (ICCS) to explore the congruence between practice and perceptions in a College of Nursing. Perkins et al., describe the process of planning and implementing focus groups, within a College of Nursing, to understand faculty, staff, and student needs specific to age inclusivity. Hart et al., detail the impact stemming from the “Excellence Across the Lifespan Champions” program, developed in partnership with the National Center to Reframe Aging to cultivate institutional culture change. Friberg-Felsted et al., explore the process of seeking accreditation through the Accreditation for Gerontology Education Council. Dr. Nina M. Silverstein will facilitate discussion on the process of assessing and developing initiatives to foster age inclusivity in higher education.

